# Hydrodynamic Evaluations of Four Mock Femoral Venous Cannulas

**DOI:** 10.21470/1678-9741-2018-0036

**Published:** 2018

**Authors:** Türker Şahin, Murat Tezer, Levent Cerit

**Affiliations:** 1 Department of Cardiovascular Surgery, Near East University, Nicosia, Cyprus.; 2 Faculty of Educational Science, Near East University, Nicosia, Cyprus.; 3 Department of Cardiology, Near East University, Nicosia, Cyprus.

**Keywords:** Cardiopulmonary Bypass, Extracorporeal Circulation, Blood Flow Velocity

## Abstract

**Objective:**

To report the results of four mock femoral venous cannulas and the hydrodynamical superiority of one of them, which is the completely punched (CP) model, upon the other three.

**Methods:**

Four simulated femoral venous cannulas (single-stage, two-stage, multi-stage, and CP model) were designed from a 1/4” x 1/16” x 68 cm polyvinyl chloride (PVC) tubing line for testing. Holes on the PVC tubes were opened by a 5 mm aortic punch. In order to evaluate the cannulas' drainage performance, gelofusine was used as fluid. The fluid was drained for 60 seconds by gravitation and then measured for each model separately.

**Results:**

Mean drained volumes of single-stage, two-stage, and multi-stage cannulas were 2.483, 2.561, and 2.603 mL, respectively. However, the CP cannula provided us a mean drained volume of 2.988 mL. There were signiﬁcant differences among the variables of the CP cannula and the other three mock cannulas concerning the drained fluid flow (*P*<0.01).

**Conclusion:**

In our study, the measured mean volumes showed us that more drainage surface area provides better fluid drainage.

**Table:** 

Abbreviations, acronyms & symbols				
ANOVAs	= Analyses of variance		MC	= Measuring cup
CP	= Completely punched		MFVCs	= Mock femoral venous cannula
CPB	= Cardiopulmonary bypass		MS	= Multi-stage
ECMO	= Extracorporeal membrane oxygenation		Pd	= Distal pressure
FVC	= Femoral venous cannula		Pp	= Proximal pressure
HVs	= Hepatic veins		PVC	= Polyvinyl chloride
ID	= Internal diameters		R1	= Reservoir 1
IVC	= Inferior vena cava		R2	= Reservoir 2
LCIV	= Left common iliac vein		RRV	= Right renal vein
LL	= Luer lock		SS	= Single-stage
LRV	= Left renal vein		TS	= Two-stage
LT	= Liquid tank		VAVD	= Vacuum-assisted venous drainage

## INTRODUCTION

In reoperative open heart surgeries, cardiac surgeons generally prefer both femoral artery and femoral vein cannulations for the safety of the operation and cardiac decompression before sternotomy^[1]^. Also, minimally invasive surgery, robotic surgery, and adult extracorporeal membrane oxygenation (ECMO) procedures^[2]^ usually require femoral vein cannulation as well. Moreover, femoral vein cannulation may be preferred in emergency departments, catheterization laboratories, and even in thoracic cases such as lung transplantation^[3]^. However, when a femoral venous cannula is needed, venous blood drainage difficulty is a key challenge for both perfusionists and cardiac surgeons.

Hypovolemia, particularly in the venous system, creates a venous blood return problem. Even in the presence of sufficient circulatory blood volume, blood may not reach the pump if there is a venous collapse^[4]^. It is known that the quality of venous drainage can be affected by pre-load volume, after-load volume, and pump design^[5]^, as well as the venous cannula's size and its design. Moreover, venous blood drainage can be affected by many reasons, such as the venous cannula's position, small sizes of vessel lumens, anatomic pathologies creating obstacles for vascular structures, massive air bloc in the venous cannula or in the tubing line, kink of the cannula or the tubing line, and obstruction of venous cannula's stages by vessels tissues or right atrial tissues, which mostly results from excessive negative pressure inside the venous tubing line^[6]^. All the above-cited reasons affect arterial blood flow and quality of perfusion. This situation obligates cardiac surgeons to insert a second venous cannula through the internal jugular vein or the axillary vein. However, some patients may, unfortunately, present with contraindications to a second venous cannula insertion from the upper site caused by a large goitre or severe neck arthrosis problems^[7]^. Therefore, a more effective femoral venous cannula is required to decrease the problems mentioned above.

The aim of this study is to find out what features should be found on femoral venous cannulas in order to provide a better drainage. In the literature, we couldn't find any experimental study including artificial venous circulation design with renal, mesenteric, and hepatic veins (HVs) lines. To perform the drainage tests under this experimental venous circulation design, we utilised four mock femoral venous cannulas (MFVCs), having different stages and different numbers of holes on the polyvinyl chloride (PVC) tube, and we performed the experiment. After some measurements and comparisons, we found that more holes on the venous cannula resulted in better venous drainage.

## METHODS

### Mock Cannulas

**Fig. 1:**
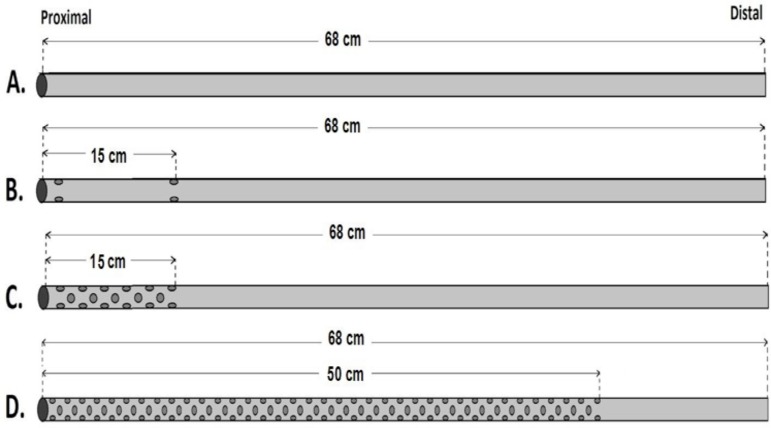
Four mock femoral venous cannulas designed from 1/4” polyvinyl chloride (PVC) tubes: single-stage cannula (A), two-stage cannula with six holes (B), multi-stage cannula with 33 holes (C), completely punched cannula (D).

There are various femoral venous cannulas available on the market today. All of them have different diameters, lengths, stages, shapes, and numbers of holes. In order to create equal conditions for the test, real cannulas were not used. To realise this condition, four different MFVCs simulating femoral venous cannulas were designed. These mock cannulas were made from PVC tube lines (RAUMEDIC-ECC) with 1/4” inner diameter and 1/16” lumen thickness. The lengths of these mock cannulas were kept the same at 68 cm. To compare the effectiveness of the number of holes, a 5 mm aortic punch was used in order to get a standardised hole diameter on the PVC tube. As indicated in Figure 1, four MFVCs were used to test and compare the performance of the different number of holes on them. These MFVCs are: model A, simulates a single-stage (SS) cannula; model B, simulates a two-stage (TS) cannula; model C, simulates a multi-stage (MS) cannula; and model D, simulates a new designed mock cannula as completely punched (CP). The SS model has no extra hole on the PVC tubing line. The TS model has six holes on the PVC line: three on the proximal tip and three 15 cm from the proximal tip of the model. The MS model has a 15 cm punched area. There are 33 holes on the proximal side of the PVC line.

The number of holes or drainage surface area is particularly significant for this study. Therefore, the last MFVC model has a 50 cm punched area with holes on the PVC line. Because of the large number of holes throughout this line, it is called a CP model. The main difference of the CP model compared with other MFVC models is that it has basically more holes on the PVC line (Figure 1).

### Mock Circulation

**Fig. 2:**
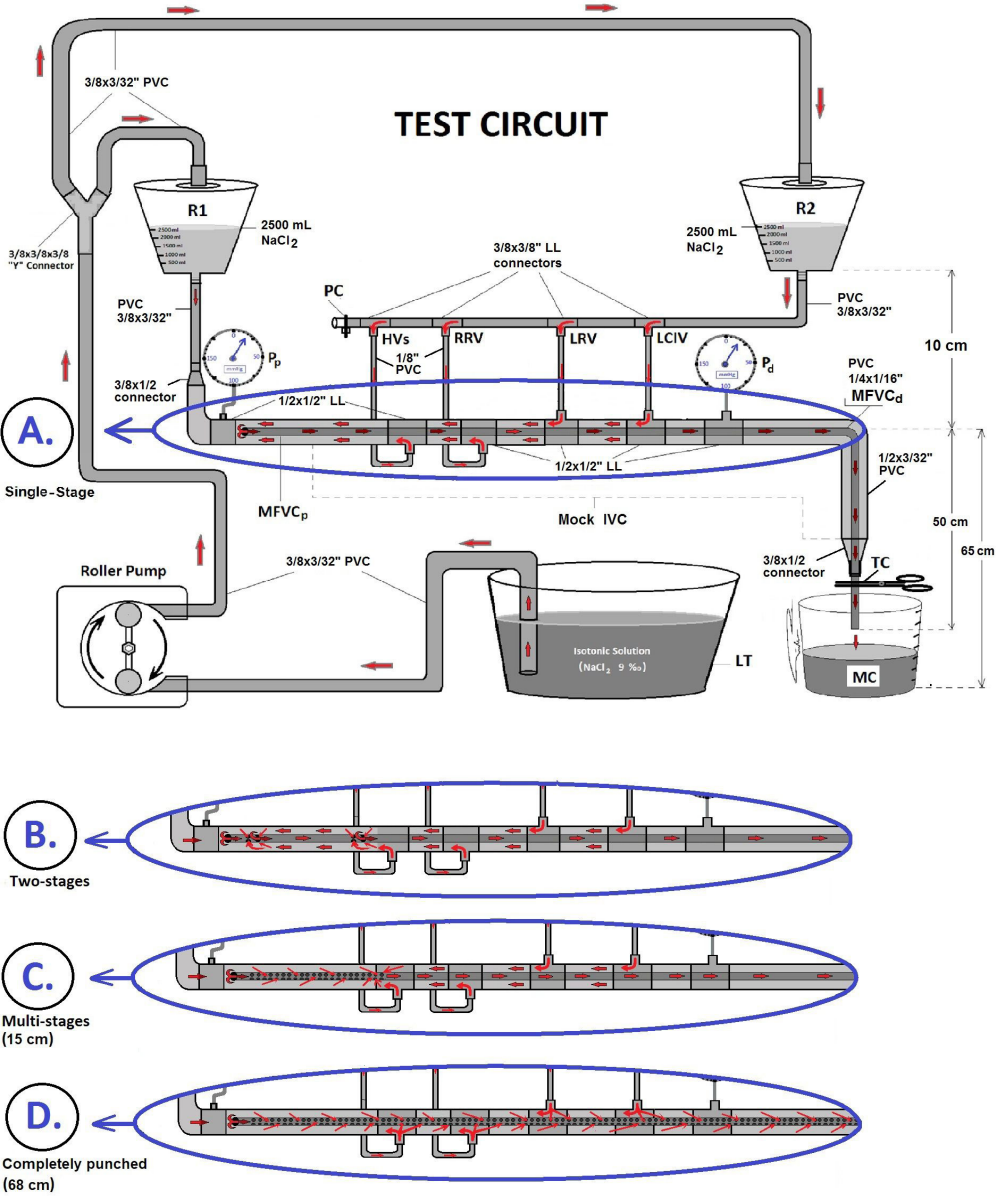
Schematic test circuit design of the four mock femoral venous cannulas, components of the mock circuit and fluid flow directions: single-stage (A), two-stage (B), multi-stage (C), completely punched (D). HVs=hepatic veins; IVC=inferior vena cava; LCIV=left common iliac vein; LL=luer lock; LRV=left renal vein; LT=liquid tank; MC=measuring cup; MFVCd=mock femoral venous cannula (distal side); MFVCp = mock femoral venous cannula (proximal side); PC=plastic clamp; Pd=distal pressure; Pp=proximal pressure; PVC=polyvinyl chloride; R1=reservoir 1 (presents the blood volume of right atrium and superior vena cava; R2=reservoir 2 (presents the blood volume of inferior vena cava and lower limps); RRV=right renal vein; TC=tubing clamp

In order to compare four different MFVCs, a test circuit model was assembled, as shown in Figure 2. Various cardiopulmonary bypass (CPB) equipments and assemblies were used, including two hard-shell reservoirs, a roller pump, a digital touch screen monitor, pressure transducers (Zyron disposable blood pressure transducer set, Point Medikal, Ankara, Turkey), reusable transducer plates (Logical®, Medex), two pressure lines (150 cm), six 1/2”x1/2” luer lock (LL) connectors, four 3/8”x3/8” LL connectors, two 3/8”x1/2” connectors, a 3/8”x3/8”x3/8” Y-connector, a tubing clamp, a liquid tank (LT), a plastic clamp band, and PVC tubes in different internal diameters (ID), such as 1/8”, 1/4”, 3/8”, and 1/2”.

Two reservoirs were positioned at the height of 75 cm and before each measurement they were filled with 2,500 mL of gelofusine solution by using 3/8” PVC lines and a roller pump (Figure 2). Reservoir 1 (R1) volume represented superior vena cava and right atrial volume. Reservoir 2 (R2) volume represented inferior vena cava (IVC) and abdominal region venous volume. The reservoirs' outlet connectors were connected by a 3/8” (ID) PVC line (50 cm) and both 3/8” PVC lines were also connected to a 1/2” PVC tubing line imitating the IVC. In order to imitate IVC well, four 1/8” PVC lines were also connected to this 1/2” PVC line. These lines, from proximal to distal, simulated HVs, right renal vein (RRV), left renal vein (LRV), and left common iliac vein (LCIV), respectively. This designed circuit was ended with a 1/2”x3/8” connector. This circuit mainly imitated the IVC's blood flow, but it also imitated the blood volume and flow of the superior vena cava and right atrium.

### Application

To evaluate the MFVCs' hydrodynamic performance, the tests were performed in a laboratory at 22^o^C room temperature, and gelofusine solution as a colloidal fluid was used in order to mimic the blood. The test circuit was completed with four MFVCs (SS, TS, 15 cm-MS, and CP), which were placed into the 1/2”x3/32” PVC line that was designed as the mock IVC. Initially, a SS mock cannula was inserted into the mock IVC. To provide priming, both reservoirs were filled with 2,500 mL of gelofusine solution withdrawn from LT by a roller pump. In order to create free gravitational force, the whole circuit and pressure lines were filled with gelofusine solution. Before test initiation, the roller pump was stopped and a tubing clamp was put on the distal tip of the mock cannula.

Pressure transducers were calibrated to “0” mmHg to ensure reliability. Whenever the tubing clamp was removed from the distal tip of this line, the countdown began at 60 seconds. The fluid in the reservoirs was drained into the measuring cup (MC) via the effect of gravity syphonic drainage. At the end of 60 seconds, the MFVC was clamped again.

Subsequently, the amount of this drained fluid was measured and recorded. After each measurement, the reservoirs were refilled up to 2,500 mL level. These measurements were repeated 34 times for each MFVC, and a total of 136 measurements were made during the entire experiment. During the drainage process, different pressures were obtained in both distal and proximal parts of the mock IVC. At the 5^th^ second, both proximal and distal pressures of the mock IVC were recorded.

### Statistical Analysis

Since the study used a within-subject design measuring four different dependent variables (SS, TS, MS, CP) and as the dependent variables had a normal distribution (*P*>0.05) according to the Kolmogorov-Smirnov test, then one-way repeated measures analyses of variance (ANOVAs) from parametric tests were performed to determine statistical differences among the groups. Also, from the *post-hoc* tests, Bonferroni tests were executed to compare the drainage flow of the four different variables for the experiment, which was repeated 34 times (n=34). A one-way repeated measures ANOVA revealed a signiﬁcant difference among the four different variables for the drained fluid flow; F_(3,99)_=8725.22, *P*<0.01. Subsequently, a one-way repeated measures ANOVA revealed a signiﬁcant difference among the four different variables for distal pressure (Pd); F_(3,99)_= 9247.20, *P*<0.01. Also, a one-way repeated measures ANOVA revealed a signiﬁcant difference among the four different variables for proximal pressure (Pp); F_(3,99)_= 180.38, *P*<0.05.

## RESULTS

**Fig. 3:**
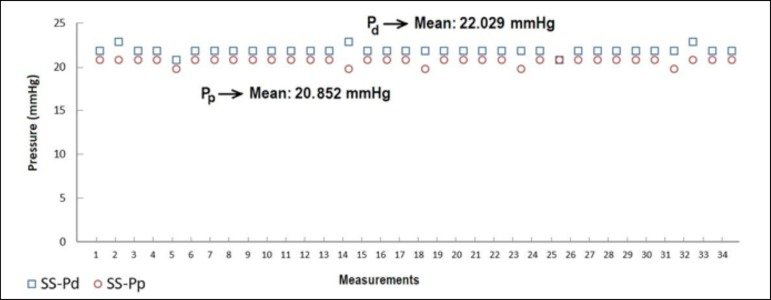
Distal (Pd) and proximal pressures (Pp) of the single-stage (SS) mock femoral venous cannula.

**Fig. 4:**
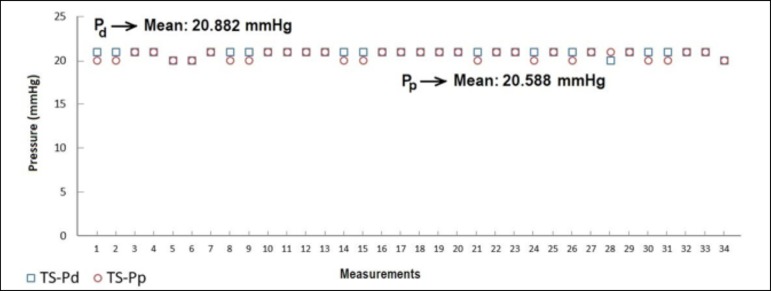
Distal (Pd) and proximal pressures (Pp) of the two-stages (TS) mock femoral venous cannula.

**Fig. 5:**
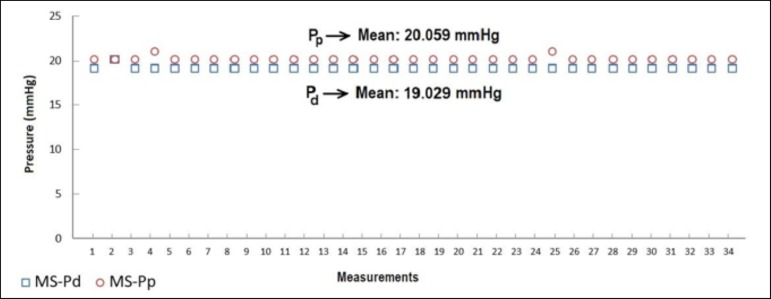
Distal (Pd) and proximal pressures (Pp) of the multi-stage (MS) mock femoral venous cannula.

**Fig. 6:**
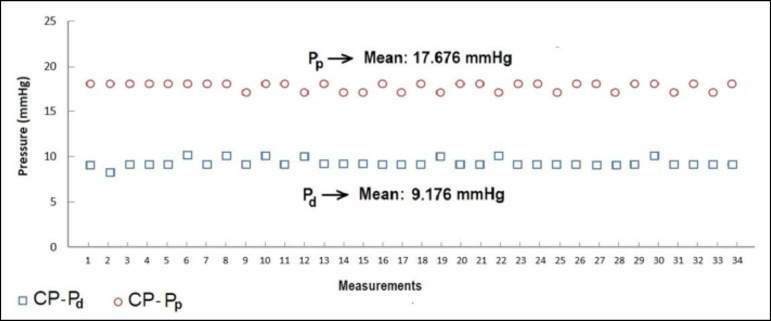
Distal (Pd) and proximal pressures (Pp) of the completely punched (CP) mock femoral venous cannula.

The data obtained from the measurements of flow amounts and pressures provide evidence to support the argument that, as the number of holes increases, the pressure markedly decreases, particularly on the proximal side. Thus, the drained volume of gelofusine increased significantly. According to the measurement results, the flow volume of the TS venous cannula was higher than that of the SS venous cannula. As expected, the volume performance of the MS venous cannula was better than those of the SS and TS cannulas. However, better drainage performances were obtained with the CP venous cannula. Figures 3 to 6 indicate pressures obtained from both distal and proximal sides of SS, TS, MS, and CP MFVCs, respectively.

**Fig. 7:**
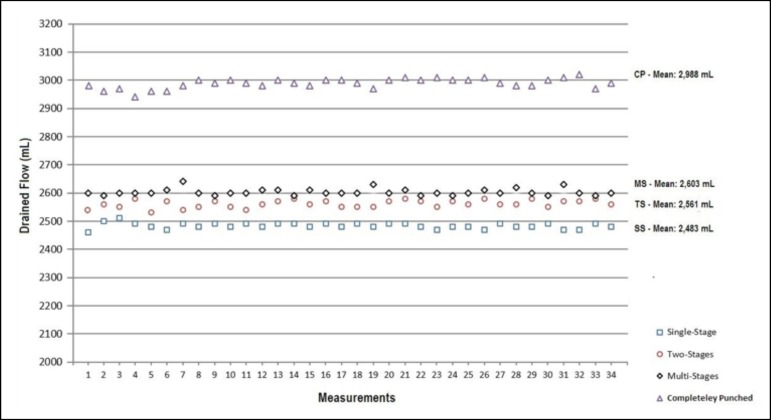
Comparison among the flow amounts provided by gravitation of four mock femoral venous cannulas in the end of 60 seconds.

According to Figures 3 to 6, while the highest Pd were measured on SS MFVC, the lowest Pd were obtained from CP MFVC. The mean pressures of the distal sides of SS, TS, MS, and CP MFVCs were 22.029 mmHg, 20.882 mmHg, 19.029 mmHg, and 9.176 mmHg, respectively. On the other hand, the highest Pp were obtained from SS MFVC, while the lowest Pp were obtained from CP MFVC. The mean pressures of the proximal sides of SS, TS, MS, and CP MFVCs were 20.852 mmHg, 20.588 mmHg, 20.059 mmHg, and 17.676 mmHg, respectively. The tests revealed that there was a statistically significant difference among the mean pressures of the proximal side of each MFVC. The results demonstrate that, as the number of holes on the MFVCs increases, both the Pp and Pd of the cannulas decrease contrarily. Pressure differences of the MFVCs resulted in different flow amounts provided by gravitation. Figure 7 indicates the differences among flow amounts of these four MFVCs. According to Figure 7, while the lowest flows were obtained from SS MFVC, the highest flows were obtained from CP MFVC. The tests revealed that there was a statistically significant difference among the mean flow amounts of each MFVC. The mean flow amounts of the MFVCs reveal that, as the number of holes increases, the flow volume increases linearly. In summary, both distal and proximal mean pressures of the four variables decreased as the number of holes on MFVCs increased.

One-way repeated measures ANOVAs indicate that the mean flow drainage is 2,483.23 mL, 2,561.17 mL, 2,603.23 mL, and 2,988.52 mL for the four dependent variables SS, TS, MS, and CP MFVCs, respectively (Table 1). The result of the Bonferroni test shows that, as the number of holes increases, the amount of drained fluid by gravitation rises significantly. On the other hand, it can be observed in Table 1 that, as the number of holes or the drainage surface area increased, the mean Pd of the MFVCs significantly decreased from 22.02 mmHg (SS model) to 9.17 mmHg (CP model). Similarly, as the number of holes increased, the mean Pp of the MFVCs significantly decreased from 20.85 mmHg (SS model) to 17.67 mmHg (CP model).

**Table 1:** Comparison among the flow amounts provided by gravitation.

Mock Cannulas	Mean Pd (mmHg)	SD*	Mean Pp (mmHg)	Mean Flow (mL)SD*	SD*
SS (1 hole)	22.02	0.38	20.85	2483.23	9.76
TS (6 holes)	20.88	0.32	20.58	2561.17	13.65
MS (33 holes)	19.02	0.17	20.05	2603.23	11.99
CP (258 holes)	9.17	0.45	17.67	2988.52	17.77

* SD=standard deviation.

CP=completely punched; MS=multi-stage; Pd=distal pressure; Pp=proximal pressure; SS=single-stage; TS=two-stage

## DISCUSSION

Venous cannula drainage improves with a high number of inlet holes^[8]^. We tried to design a CP MFVC having more holes on it. In our study, we found that the CP MFVC model performed better for fluid drainage under the same test conditions than each mock cannula. Our study revealed that a greater drainage area (number of holes on the tube) is the key factor for a better fluid drainage amount.

In fact, the main reason behind the difficulties experienced with venous blood drainage is the smaller size of the femoral vein. In such situation, the vein's diameter is generally not suitable for insertion of a larger venous cannula for full perfusion. To overcome this problem and to achieve full perfusion flow during a CPB procedure, perfusionists generally must use a centrifugal pump on a venous line or use negative pressure (-20 to -40 mmHg), which is called vacuum-assisted venous drainage (VAVD). Unfortunately, these techniques create more complex connections and may increase complications during CPB. When applying higher negative pressure into the venous line^[9,10]^, gas microembolus will increase. Also, assisted venous drainage may lead to air incoming to the venous bloodstream. Furthermore, the higher negative pressure in the venous line will create higher shear stress and haemolysis. To avoid such complications and to prevent vein collapse, additional monitoring is also required^[11,12]^. Under these conditions, the design of femoral venous cannulas is highly important.

The four different mock cannulas shown in Figure 7 were tested regarding the relationship between the number of holes on their tubing walls and the amount of fluid drainage. In fact, there were many factors affecting fluid drainage and creating handicaps during these tests, such as the position of the lines, position of the MFVCs that were inserted throughout the inside of the mock IVC, level of the distal tip of the MFVCs, starting or stopping fails on chronometry, fluid's viscosity, test room's temperature, etc. Obviously, all these variables may affect liquid dynamics and measurements of the fluid amount. Nonetheless, this test design simulating femoral venous cannula and human IVC provided the facility to measure and compare four MFVCs, not only regarding their Pd and Pp inside but also their provided amount of fluid.

In fact, the venous blood return towards to the right atrium is a result of the pressure difference between the mean circulatory filling pressure and the pressure in the right atrium. All these pressures and inner surfaces of the vessels create intravascular resistance against the blood flow, and they contribute to the creation of shear stress. In this experimental circuit, there was no such contrary pressure making resistance to the gelofusine solution in the tip of the MFVC. This peculiarity of the artificial circuit provided a very similar condition regarding venous blood drainage to the venous reservoir during open heart surgery.

Many factors, such as the diameters and shapes of the cannulas, connections and structures of the MFVCs, fluid's temperature, test room's viscosity, and velocity of the fluid used in the test circuit, are influential on shear stress. We estimate that all the above-cited factors affected the shear stress and the flow volumes drained by gravitation in the tubes. Thus, they all contributed to create different pressures in the distal and proximal sides of the MFVCs. These factors create shear stress differences among the MFVCs, consequently causing flow differences.

The possible venous blood contribution for the VCI blood flow mainly comes from the HV, RRV, LRV, LCIV and right common iliac vein. If a femoral venous cannula is inserted into the right femoral vein towards the right atrium, the cannula itself will form a barrier to the venous blood coming from iliac veins and renal veins. In order to simulate this scenario, four 1/8” PVC lines were connected to the mock IVC in the test circuit (Figure 2). Through this design, different drainage capabilities of four MFVCs were evaluated and compared. In conclusion, this study demonstrated that the CP MFVC provided better fluid drainage by free gravitation than the other MFVC models.

### Limitations

In the clinical application, all the holes of the CP femoral venous cannula (FVC) must be inserted completely into the venous system, therefore different sizes of CP FVCs must be kept ready in the operating theatre and they must be selected according to the length of the patient.

This study may be criticised regarding its simulation efficiency and its fidelity of MFVC or mock IVC. In fact, our objective was not to achieve the perfect fidelity of the circulatory and vascular systems. The aim of this study was limited to created standard conditions for the MFVC tests. Yet, many more variations, such as other fluids (hetastarch 6%, plasma, and even whole blood with different hematocrit) in different rooms and fluid temperatures, precise chronometry usage, the fidelity of vascular response and flexibility, *in vivo* studies in animal laboratory, etc., may be considered throughout the test circuit design in future studies about drainage performance of CP MFVC.

## CONCLUSION

When we compared the results, they suggested that the CP MFVC model provided better fluid drainage under the same test conditions than other MFVC models. While the CP MFVC shows a reduced pressure for distal and proximal sides of the cannula, it drained much more fluid. Our study may shed light on *in vivo* studies, which need to be addressed in the future. This novel cannula design aims to provide better and simply venous blood drainage from femoral vein.

**Table:** 

Authors' roles & responsibilities	
TS	Conception and design of the work; acquisition of data; analysis and interpretation of data; drafting the paper; revising the work; approval of the final version
MT	Analysis and interpretation of data; drafting the paper; revising the work; approval of the final version
LC	Analysis and interpretation of data; drafting the paper; revising the work; approval of the final version
