# ERRATUM

**DOI:** 10.21470/1678-9741-2018-0036e

**Published:** 2019

In the article "Hydrodynamic Evaluations of Four Mock Femoral Venous Cannulas", published in the Brazilian Journal of Cardiovascular Surgery 33.5, pages 435-442, the correct title of one of the co-authors is Murat Tezer^2^, PhD and not Murat Tezer^2^, MD.

